# Concordance between European medicine agency good clinical practice inspections and medical literature: a meta-research survey

**DOI:** 10.1186/s12916-025-04499-9

**Published:** 2025-12-03

**Authors:** Alexandre Terré, Ondine Becker, John P. A. Ioannidis, Florian Naudet

**Affiliations:** 1 Department of Internal Medicine, Ambroise Paré Hospital, Boulogne-Billancourt, France; 2 Faculté de Médecine, Sorbonne Université, Paris, France; 3 Medical Oncology Department, European Hospital Georges Pompidou, Paris, France; 4 Departments of Medicine, of Epidemiology and Population Health, and of Biomedical Data Science, and Meta-Research Innovation Center at Stanford (METRICS), Stanford University, Stanford, CA 94305 USA; 5 IRSET (Institut de Recherche en Santé, Centre of Clinical Investigation of Rennes), University of Rennes, CHU Rennes, INSERM, Environnement Et Travail)-UMR_S 1085, CIC 1414, Rennes, France; 6 Institut Universitaire de France (IUF), Paris, France

**Keywords:** EMA, EPAR, Meta analyses, Post publication peer review, Good clinical practice inspections

## Abstract

**Background:**

Health authorities, such as the European Medicines Agency (EMA), inspect clinical sites performing clinical trials and occasionally find evidence of substantial departures from Good Clinical Practice (GCP). However, if the findings are not transposed in the medical literature, publication of those trials may convey inaccurate messages.

**Methods:**

**Objective:**

to describe the differences between EMA GCP inspection reports and medical literature on drugs with withdrawn or refused applications.

**Design:**

a retrospective study comparing studies included in European Public Assessment Reports (EPARs) and the corresponding articles published in the medical literature.

Data sources—we screened all EPARs released by the EMA from inception to April 2024 for drugs that were refused or had a withdrawn application.

Data extracted*—*two reviewers independently gathered information on the GCP inspections and their findings. The reviewers checked related publications for mentions of the inspection and any subsequent correction, retraction, or expressions of concern related to its findings.

Main outcome measures*—*the main outcome was the mention of the GCP inspection findings in the publication of the inspected studies. We also assessed whether there was any mention of these findings in a correction, retraction or expression of concern.

**Results:**

Out of 285 EPARs screened, 57 (20%) mentioned a GCP inspection. Fifty-eight distinct studies with inspections had 61 publications. In most of the cases (*n* = 47, 77%), the study was published before the EPARs. Only 1 publication (2%) addressed the inspection findings. Moreover, there were no corrections, retractions or expressions of concern related to inspection findings. Among the 61 publications, 26 (43%) were related with 24 distinct studies that had an inspection that casted doubts on data reliability, but none mentioned the inspections at or after the time of publication.

**Conclusions:**

This meta-research survey indicates that health authorities’ GCP inspections are not reflected in the medical literature, even when the inspections have put the data reliability in doubt. This study raises the question of responsibility for any corrections that may be necessary in medical literature following the publication of EPARs.

**Supplementary Information:**

The online version contains supplementary material available at 10.1186/s12916-025-04499-9.

## Background

Clinical trials are essential sources of information for healthcare professionals and public health policymakers. Due to the significant impact that their publication in medical literature can have on patient care, it is crucial that this information is trustworthy. Peer review prior to publication serves to identify and rectify errors, thereby ensuring the reliability of the literature. However, peer review has its limitations [1, 2]. It often faces limited resources, and external experts may struggle to comprehensively address all potential issues within the constrained time frame. Sometimes, criticisms or comments by the reviewers may escape this step, leading to the publication of clinical trials with errors [3–5].

Health authorities such as the US Food and Drug Administration (FDA) and the European Medicines Agency (EMA) occasionally conduct Good Clinical Practice (GCP) inspections at clinical study sites, providing an in-depth evaluation of crucial steps of clinical trials, such as conduct, data collection and analysis. The detail provided by such investigations provides material that exceeds what medical journals can offer with peer-review. The report of these inspections, when identifying deficiencies, is crucial and should be known by the end-users. However, it seems that these inspection reports are disconnected from the evidence published in the medical literature. In 2015, Seife identified 57 clinical trials resulting in 78 publications where a GCP inspection by the FDA found deviations from GCP recommendations that could cast doubt on the quality of the information presented in the corresponding publications [6]. Yet, in 2015, at the time of Seife’s publication, 96% of the articles he identified did not mention these inspections either themselves or through an expression of concern or erratum [6].

This question has not been examined through clinical trials assessed by the EMA. Unlike the FDA, the EMA does not directly publish GCP inspection reports. However, these can be referenced in the European Public Assessment Reports (EPAR). EPAR are issued by the Committee for Medicinal Products for Human Use (CHMP). Unlike trial monitoring, EMA’s GCP inspections are usually performed after the trial completion, when the sponsor submits an application for a market authorization. EMA’s GCP inspection can be triggered by specific concerns found in the application or are sometime routine inspections. They are decided by the EMA and are not country specific. Inspections are performed by inspectors from countries members of the EMA; there are at least 2 inspectors from different countries. Inspectors’ qualifications may vary from country specification, but the inspection process of the EMA is a standard procedure [7].

It is not mandatory in the EMA rules to present the GCP inspection in the EPAR. However, when the outcome of a GCP inspection influences the CHMP’s decision to approve or deny a marketing authorization [8], it is usually presented. The total number of inspections performed each year by the EMA is publicly available on the EMA web site (example for the year 2018 [9]. It displays the total number of site inspections (i.e. each inspected site, for example, for an application inspection of 2 trials sites, and the sponsor will be counted as 3 inspections). It is not possible to access the percentage of market application that were subject to inspection. From the annual activity reports, one can see that GCP inspections are not uniformly distributed during our study period as the number of inspections declined during the COVID-19 pandemic and slowly rose back after that.

The objective of our study was to determine if the EPARs for drugs submitted to the EMA contain information from GCP inspections that contradicts the medical literature data. To increase the chances of identifying GCP inspections that raised concerns, we focused on marketing applications that were either refused or withdrawn.

## Methods

The methods of this descriptive study were specified in advance in a protocol registered on the Open Science Framework on 31 st July 2024 (https://osf.io/pa9fq/ [10]). This report provides details about any deviations from the initial plan. As no reporting guideline exists for such a meta-research survey, we relied on PRISMA [11] which we considered as the most appropriate.

### Eligibility criteria and information sources

We included EPARs published from the 17th of July 2000 (date of publication of the first EPAR) up to the 1 st of April 2024, concerning drugs refused or for which the marketing application has been withdrawn. Among those EPARs, any study with a GCP inspection was included. The EPARs are labelled under the commercial name of the drug on the EMA website, thus here we use this name in our article and for EPARs citations.

### Search strategy

The assessments of the CHMP are publicly available on the EMA website, under the name of European public assessment reports (EPAR) [12]. Using the search function on the EMA website, we searched the published assessments reports using the filters “human”, “Medicines”, “Withdrawn application”, “refused”.

For each identified assessment, we searched the available documents on the EMA website for mentions of a GCP inspection conducted by the EMA by searching the terms “Good clinical practice” or “GCP” used in these reports. We preferably used the document “EPAR—Public assessment report”.

### Selection and data collection processes

Screening of EPARs and data collection of verbatim quotes were done independently by two authors (AT and OB). Discrepancies were first resolved through consensus before being arbitrated by a third author (FN).

### Data items

#### Assessment report data

For each identified EPAR, we recorded the opinion date, the commercial name of the drug, the international non-proprietary name, the application status and whether a GCP inspection conducted by the EMA was mentioned.

When a GCP inspection was identified, we extracted whether it was described and if deviations were mentioned in the EPAR. For each study subject to a GCP, we noted if the results of the inspection impacted the efficacy criterion, the safety/tolerance profile of the drug or an ethical rule. The deviations observed by the EMA are classified as “critical”, “major” or “minor”. Precise definition of critical, major or minor findings can be found in the Additional file 1: supplementary methods [7].

We extracted information following this classification by using the exact terms employed in the report. We extracted the number of “critical” deviations per GCP inspection. If the EPAR detailed the verbatim elements of the GCP inspection influencing its decision, these elements were collected in free text. Finally, we looked at the conclusions issued by the CHMP concerning the impact of deviations eventually observed on the results presented. To clarify this last aspect, we had to refine data analysis process during our study because the classification of the findings as “critical”, “major” or “minor” was frequently missing or was not related to the reliability of the data as assess by the CHMP. Using the verbatim text presented in the EPAR, we extracted the opinion of the EMA on the effect of the GCP findings on the reliability of the data. The findings were classified as “impacting the reliability of the data”, or “not impacting the reliability of the data”. If the opinion was not described, or ambiguous, the opinion was classified as “unknown”.

#### Subsequent publications

For each mention of a GCP inspection, we searched if the corresponding studies were published in a peer-reviewed medical journal. The search strategy is described in the Additional file 1: supplementary methods. For each identified publication, we examined references to the GCP inspection and its conclusions. We also searched for corrections, retractions or expression of concerns mentioning the GCP findings on PubMed and on the journal website. This outcome was added during the research because many articles were published before the EPAR, so the initial version of the article was not expected to mention the GCP inspection. During the study, a search was added on the PubPeer website [13] for any comments posted about the articles.

#### Dissemination in meta-analyses, reviews, and citation count

For each identified publication, we used the Web of Science search engine to collect the citation count of each study and specifically study the number of meta-analyses citing them. This was done between the 2nd of January and the 5th of February 2025.

#### Detailed inspection reports

To obtain details of GCP inspections revealing critical or major deviations and resulting in a scientific publication, we requested the complete GCP inspection report from the EMA. According to the EMA policy, inspection reports are accessible to the public [14]. We collected the date of request, the date of answer and the answer we received.

#### Outcomes

The primary outcome was the mention of the deviation or of the EMA GCP inspection in the publication. This definition had to be enhanced by incorporating information on corrections, retractions or expressions of concern related to the GCP findings. The secondary outcomes were (1) description of the deviations in the EPAR, both qualitatively and quantitatively; (2) discordance between the deviations described in the EPAR and the publication; (3) description of publications in terms of journal and date; (4) dissemination of the studies with data reliability issues identified by the EMA in journals and meta-analyses; and (5) comments on PubPeer about the GCP inspection (this outcome was added during the study).

### Analysis

We described quantitative variables using means (± standard deviations, SD) or medians (interquartile range, IQR), as appropriate. We described qualitative variables using numbers and percentages. Verbatim text is presented to describe findings of the GCP inspections.

### Open access

Data and code to reproduce the analysis are available on the Open Science Framework (https://osf.io/pa9fq/files/osfstorage [10]). EPARs used for the study are cited via the EMA link.

## Results

### Selection of EPARs, GCPs, published articles

**Fig. 1:**
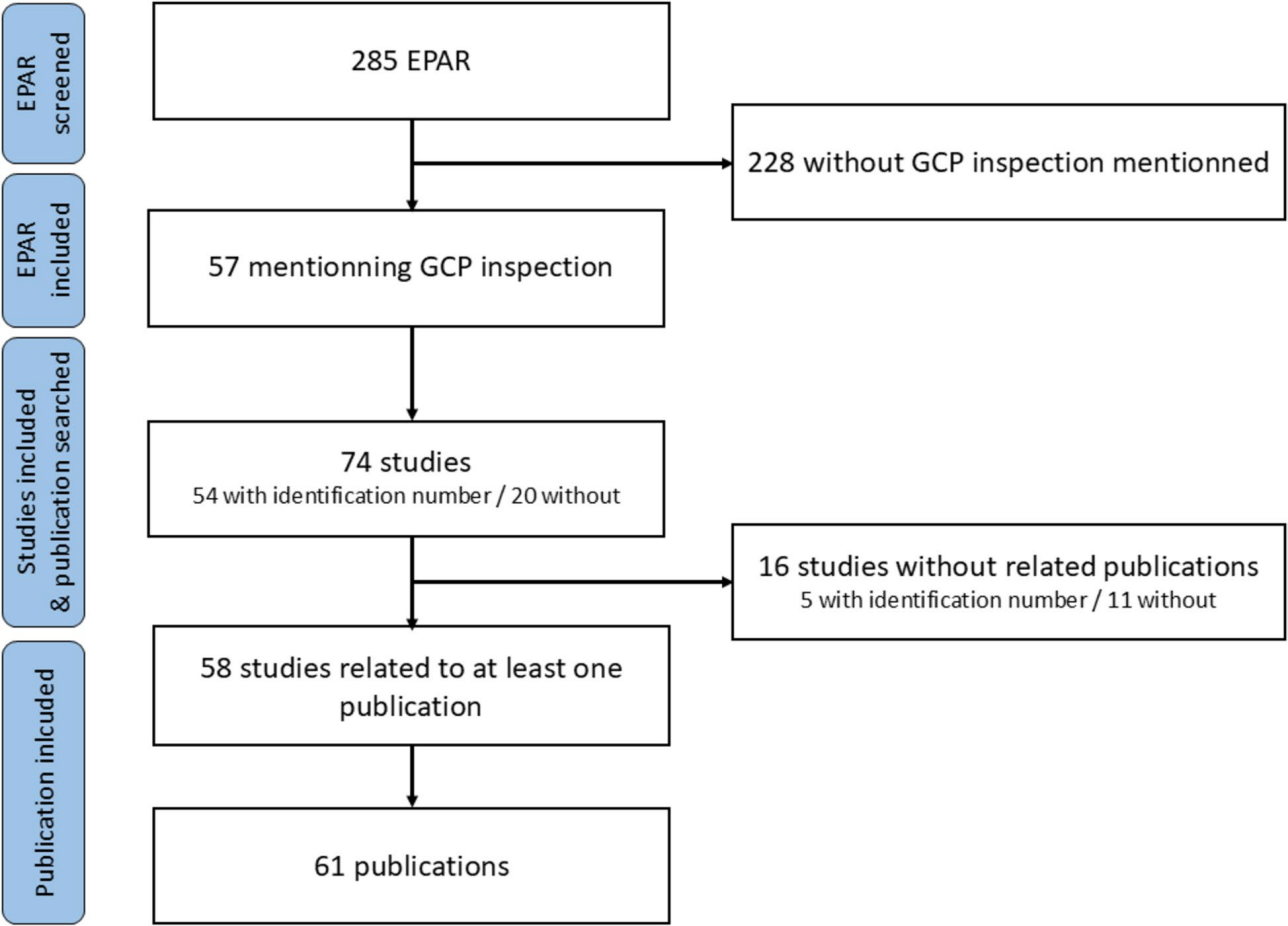
Flow chart of the study

We screened 285 EPARs of refused or withdrawn applications. Fifty-seven (20%) of those were included [15–71] as they mentioned a GCP inspection. Description of the EPARs can be found in Table 1. Those 57 EPARs referred to 74 different studies, 54 (73%) with a registered identification number (NCT or EudraCT). Among the 74 studies, 58 (in 45/57 EPARs) could be related to at least one publication. Among those, 52/58 studies (90%) were related to only one publication, and 6/58 (10%) to 2. Three publications reported results of 2 studies. This resulted in a total of 61 publications [72–132]. Figure 1 describes the selection process.

**Table 1:** Description of the inspected EPARs

Characteristics	Inspected EPARs (*n* = 57)
**Year of publication**	
Median	2014
Range	2007–2023
**Type of application**	
New drug	49 (86%)
Generic	4 (7%)
Biosimilar	4 (7%)
**Orphan drugs**	16 (28%)
**Status**	
Refused	17 (30%)
Withdrawn application	40 (70%)
**Medical specialty**	
Oncology	14 (25%)
Hematology	9 (16%)
Infectious diseases	7 (12%)
Ophtalmology	6 (11%)
Neurology	5 (9%)
Rheumatology	4 (7%)
Genetic diseases	3 (5%)
Nephrology	3 (5%)
Pneumology	3 (5%)
Cardiology	1 (2%)
Endocrinology	1 (2%)
Surgery	1 (2%)

### Description of the deviations in the EPARs

**Fig. 2:**
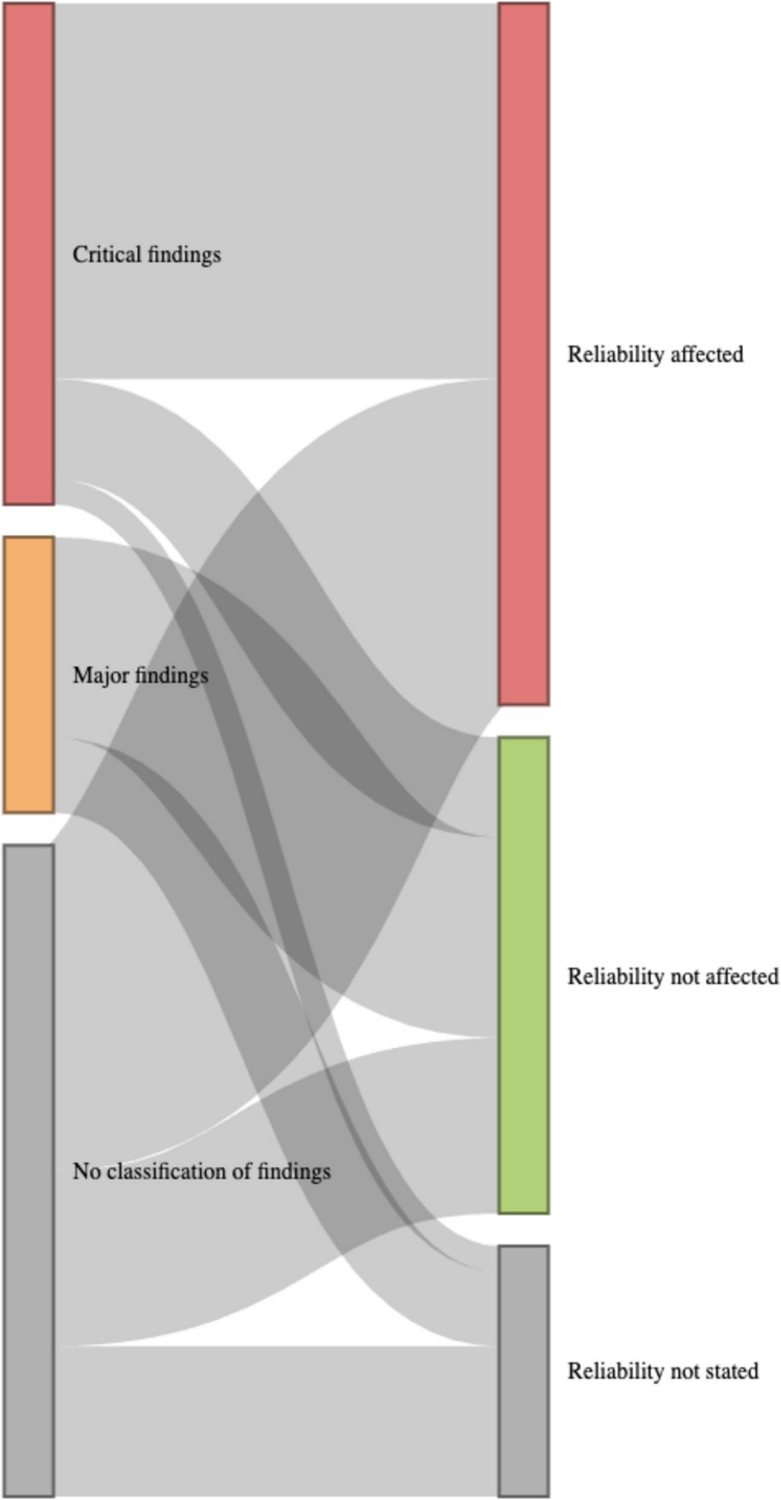
Sankey diagram showing the classification of Good Clinical Practice inspection in relation to reliability assessment by the Committee for Medicinal Products for Human Use. The data and code necessary to replicate the figure can be accessed through the Open Science Framework (https://osf.io/pa9fq/)

About 26/57 EPARs (46%) did not specify whether the GCP inspection findings were classified as critical, major, or minor. Moreover, 20/57 (35%) EPARs reported “critical” findings during the GCP inspections, and 27/57 (47%) reported “major” findings (presence of critical and major findings are not exclusive). As described in 34/57 (60%) EPARs, the deviation affected the efficacy outcome in 26/34 (76%), the safety in 20/34 (59%) and ethics in 4/34 (12%) (deviations can be observed in multiple aspects of the study within an EPAR). Among the 57 EPARs, the GCPs findings were classified as “impacting the reliability of the data” (in at least on study mentioned in the EPAR) for 28/57 (49%) and “not impacting the reliability of the data” for 19/57 (33%). For 10/57 (18%), details were insufficient to judge the impact on reliability of the data from included studies. As the EMA defines critical findings as “conditions, practices or processes that adversely affect the rights, safety or wellbeing of the subjects and/or the quality and integrity of data. Critical observation is considered totally unacceptable” we were surprised that some EPARs mentioned critical deviations but still concluded that the data were reliable. Figure 2 describes the correspondence between EMA’s classification and the impact on data reliability. This figure highlights that the GCP inspection findings classification is not sufficient to assess the data reliability. Information on the number of deviations per inspection was missing in 39/57 (68%) EPARs. In the EPARs mentioning critical deviations (*n* = 20/57; 35%)), the number of critical deviations was described in 6/20 (30%). A median of 5 (IQR 3.25–9.75) critical deviations were reported. Those results are summarized in Additional File 2: Table S1.

### Mention of the deviation and/or of the EMA GCP inspection in the published record

**Fig. 3:**
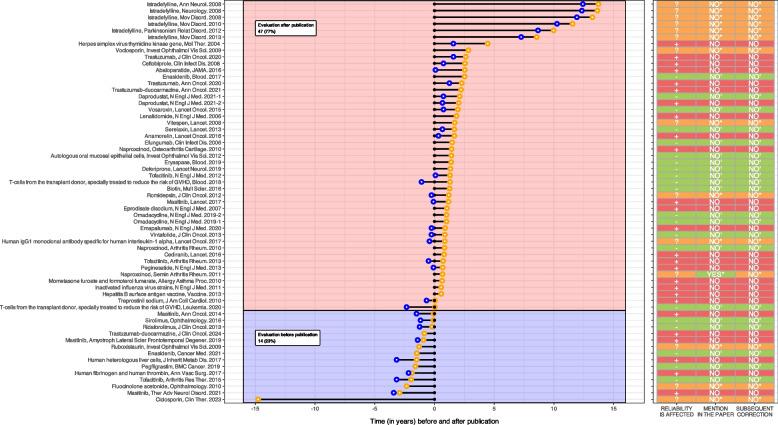
Chart showing the date of the inspection report (blue points), the date of the publication of the European public assessment report (orange points), and the date of the publication in a medical journal (black points), along with EMA’s evaluation regarding the reliability of the findings, mention in the publication, and subsequent correction, retraction, or expression of concern- = data reliability non affected;  +  = data reliability affected;? = unknown impact of the inspection’s findings on data reliability; ‘ = no mention of the findings expected;* = Findings that may or may not be relevant to report, as it was unclear whether the reliability was affected. The data and code necessary to replicate the figure can be accessed through the Open Science Framework (https://osf.io/pa9fq/)

The information on publications is available in Additional File 2: Table S2. Regarding our primary outcome, only 1/61 (2%) mentioned GCP inspection findings in the publication. This article discussed naproxcinod for knee osteoarthritis. The EPAR excluded data from a site due to GCP breaches, including lack of informed consent and recruitment of site personnel or their associates [30]. The publication mention: “1 site excluded due to quality issues, and 1 patient excluded having been randomized at 2 sites” [118]. As detailed in Fig. 3, the corresponding EPARs were released in the public sphere after the article publication for 47/61 (77%) publications. Among those, the GCP inspection occurred after publication for 17/45 (38%) and occurred before the publication in 8/45 (18%). For the remaining 20/45 (44%), the date of the GCP inspection was unknown. We found no corrections, expressions of concern, retractions, related to the GCP inspection findings.

For 21 publications (36%), the EPAR indicated that the GCP inspection did not affect the reliability of the data. For 14 publications (20%), the impact on data reliability was not described or was ambiguous. For 26 publications (43%), the EPAR indicated that GCP findings raised concerns about data reliability. The results are summarized in Fig. 3. Only 2/61 (3%) publications [96, 104] had comments on PubPeer concerning the CGP inspection.

Description of the 16/74 (22%) unpublished studies can be found in Additional file 2: Table S3.

### Deviations, publications and dissemination of studies with data reliability issues

For the 26 studies with data reliability issues, the verbatim text of the EPAR mentioning the main findings and the journals in which those studies were published are presented in Table 2. The only journals with multiple published articles of this category were *The New England Journal of medicine* (*n* = 6, 23%), *The Lancet* (*n* = 2, 8%), *Annals of Oncology* (*n* = 2, 8%). Those studies with reliability issues were published between November 2004 and October 2024 and had a median citation number of 135 (IQR 46; 308.5) and a median citation number by meta-analyses of 5 (IQR 1; 9.5).

**Table 2:** Studies with inspection findings affecting the data reliability. Selected elements of the description of the good clinical practice inspection are provided

*Drug* (International non-proprietary name) and therapeutic indication	Journal, year and (reference)	GCP inspection description in the public assessment report	Citations	
			**Total**	**MAs**
*Ceftobiprole* in the treatment of complicated skin and soft-tissue infections in adults	Clinical Infectious Diseases, 2008, [108]	“The GCP inspectors recommended the exclusion of the data from 2 out of the 4 investigator sites inspected”. “As each round of inspection or audit has led to the exclusion of additional sites, the overall conduct of the trial is questionable and it can no longer be concluded that the conduct of the trial did not undermine its ability to distinguish effective treatments from less effective treatments”. “The GCP non-compliance observed in the conduct of the two pivotal clinical trials does not allow the conclusion that the currently available safety database for ceftobiprole is comprehensive and truly reflects all potentially adverse events due to treatment with the compound”	208	12
*Masitinib* in the treatment of malignant gastrointestinal stromal tumour (GIST)	Annals of oncology, 2014, [72]	“The inspection revealed critical deficiencies, which raise concerns about the efficacy and safety data reported in the CSR of the pivotal trial AB07001 for investigator sites 01 and 03 (22 patients’ records out of 43 patients in total in the trial representing 51% of the patients in this pivotal trial)”. Inspectors cannot recommend that the presented data and CSR is accepted by the CHMP or used for further assessment	54	6
*Human heterologous liver cells*, in the treatment of urea cycle disorders	Journal of Inherited Metabolic Disease, 2017, [99]	“The inspection revealed critical and major findings related to study design, conduct and oversight of the study, safety reporting, IMP handling, monitoring of the trial and informed consent handling”. “The inspection report concluded that the conduct of the trial was GCP non-compliant and recommended the data collected should not be used in the context of the marketing authorisation application”. “Furthermore, the results of the GCP inspection raised concern as regards the completeness and reliability of the AE/SAE (serious adverse events) that were reported”	14	0
*Anamorelin*, in the treatment of anorexia, cachexia or unintended weight loss in patients with non-small cell lung cancer	The Lancet Oncology, 2016, [126]	“The inspections revealed several flawed GCP findings, including significant deficiencies in relation to the availability of source data at the investigator sites, availability of the clinical database, collection and reporting of safety data as well as the investigational medicinal product delivery”. “The inspection report concluded that safety data reported in the clinical study reports cannot be recommended for assessment”. “The GCP inspection conducted in for this application revealed that concomitant medication – other than for tumour treatment—was not systematically collected. A potential impact of, e.g. systemic corticosteroids on weight gain could therefore not be assessed”	415	9
*Masitinib*, in the treatment of systemic mastocytosis	The Lancet, 2017, [96]	“The following inspection findings were identified during the two clinical site inspections: Missing adverse events from the study report, mistakes in the reporting of abnormal laboratory results as adverse events, failures in the handling of serious adverse events, lack of a database for SAEs”. “the GCP inspection has found that the blinding was compromised during the trial” “an incorrect version of the AFIRMM V2 questionnaire was used during the first months of the study, and occasionally thereafter, however the data obtained were pooled with the other data, confusing their interpretation”. “The accumulation of critical and major inspection findings, affecting all aspects of the trial, seriously question the reliability of the trial data”	93	2
*Tofacitinib*, in the treatment of rheumatoid arthritis	Arthritis and Rheumatism, 2013, [129]	“However, the major and critical findings suggested some deficiencies in the presentation of data in the CSRs yielding the inspectors to request additional reanalyse”. “There was concern that the statistical methods employed to handle patients who discontinued from the randomised treatment may overestimate the treatment effect”. “Data from patients at Sites 1048 and 1174 were excluded from the FAS”	455	42
*Masitinib,* in the treatment of amyotrophic lateral sclerosis	Amyotrophic Lateral Sclerosis and Frontotemporal Degeneration, 2019, [104]	“The nature and of the findings, the systematic deficiencies observed, the massive number of protocol deviations which translated into a general lack of adherence to the protocol, the lack of global investigator meetings to harmonise procedures, and the suboptimal quality of management by the sponsor and CROs involved, all have a negative impact on data reliability”. “One of the remarked issues in GCP inspection was the fact that similar reasons for withdrawal were registered differently, thus leading to different imputations in the final analysis”. “It was concluded that the data obtained at the inspected sites are not trustworthy enough to support the submitted marketing authorisation application for masitinib, and seriously affect the credibility of the data in the whole application”	135	1
*Masitinib,* in the treatment of amyotrophic lateral sclerosis	Therapeutic Advances in Neurological Disorders, 2021, [103]	“The nature and of the findings, the systematic deficiencies observed, the massive number of protocol deviations which translated into a general lack of adherence to the protocol, the lack of global investigator meetings to harmonise procedures, and the suboptimal quality of management by the sponsor and CROs involved, all have a negative impact on data reliability”. “One of the remarked issues in GCP inspection was the fact that similar reasons for withdrawal were registered differently, thus leading to different imputations in the final analysis”. “It was concluded that the data obtained at the inspected sites are not trustworthy enough to support the submitted marketing authorisation application for masitinib, and seriously affect the credibility of the data in the whole application”	38	0
*Abaloparatide*, in the treatment of osteoporosis	JAMA, 2016, [100]	“critical findings were identified relating to patient’s inclusion in the clinical trial, the quality system of the sites and the availability of documents requested during the inspection”. “Due to the critical findings observed at two sites in Czech Republic, the inspectors concluded that the data from these sites cannot be reliable, and recommended to exclude them when assessing the marketing authorisation application. The exclusion of these two study sites in the single pivotal study resulted in a reduction of the total study population by 16% from 2463 to 2070 participants”. “A significant and clinically relevant efficacy of abaloparatide compared to placebo on non-vertebral fractures has not been established [log-rank *p* = 0.3675; HR (95% CI) 0.74 (0.38, 1.43)]”	571	25
*Emapalumab,* in the treatment of primary haemophagocytic lymphohistiocytosis (HLH) in children under 18 years of age	The New England Journal of Medicine, 2020 [95],	“Inspection resulted towards important critical and major findings related to the patient’s rights and the data integrity”. “Deviations regarding to informed consents as for example, no translated informed consent available; the collection of more medical data and storage of patient samples than was foreseen by the protocol or informed consent, the transfer to the sponsor of personal data like race, ethnicity, and sometimes patient’s identity”. “The Data Monitoring Committee was not independent as a sponsor representative was always present”. “The critical findings found at the Sponsor site, impacted the whole clinical trial. In this case it negatively affected the data collection, data handling, the planning of the transition of the phase II to a phase III trial, statistical analyses, etc.” “The study NI-0501–04 was not considered GCP compliant and the integrity of the data is impaired”	319	0
*Herpes simplex virus thymidine kinase gene*, in the treatment of patients with operable high-grade glioma	Molecular Therapy, 2004, [87]	“The NAM regulation and the principles of good clinical practice were not followed in all aspects of the study 903”. “ Although the study 903 was systematically performed, a valid amended protocol or other valid amendment document was missing for several years during the conduct of the study”. “Although the clinical data collected in this study could be verified, the concerns with respect to data quality remain. CRFs were created retrospectively. Thus, only information available in the patients’ records was collected retrospectively”. “However, there is a large amount of information that is either poorly documented or is lacking in many patients”	298	5
*Eprodisate disodium*, in the treatment of amyloid A amyloidosis	The New England Journal of Medicine, 2007, [80]	“A Cox analysis was performed which was not described in the statistical analysis plan, with this analysis subsequently presented as the principal evidence of product efficacy”. “The sponsor could not ascertain that the blind was properly maintained throughout the trial”. “The explanations and the documentation provided by the applicant were insufficient to guarantee complete reassurance that the randomization list was not used for the purpose of an interim analysis”. “Despite additional information and due to a deviation related to the blinding process and lack of documentation, the EMEA cannot be certain that the decision to change the primary efficacy endpoint was not datadriven”. “The originally primary endpoint, which included assessment of changes in nephrotic syndrome and gastrointestinal symptoms showed no difference between eprodisate and Kiacta (*p* = 0.79)”. “A sensitivity analysis of the primary outcome imputing all. discontinuations to the ‘worse’ category demonstrated the lack of robustness of the primary outcome (*p* = 0.340)”	162	1
*Lenalidomide*, in the treatment of anaemia due to myelodysplastic syndromes	The New England Journal of Medicine, 2006, [94]	“incomplete reporting of transfusion and non-compliance to transfusion rules which could have led to an overestimation of efficacy”. “Non-compliance to transfusion rules predefined by the protocol has been observed in roughly 40% of patients. In addition, in these patients who were not completely followed by the investigators, additional transfusions prescribed outside the study might have impacted on efficacy results”. “The applicant provided updated data on deaths and AML occurrence which are further detailed and discussed in the safety section […] substantially higher incidence of Grade 3 and 4 thrombocytopenia and neutropenia than initially reported”. “Major findings were highlighted during the GCP inspection of one investigational site and these observations could affect the reliability of the collected safety data, in particular the rate of AML transformation”	1009	6
*Treprostinil sodium,* in the treatment for patients with pulmonary arterial hypertension	Journal of the American College of Cardiology, 2010, [98]	“Based on the Agency inspections at two different trial sites, the pivotal TRIUMPH study is considered non-GCP compliant and therefore not in accordance with requirements of Directive 2001/83/EC”. “The conclusion of non-compliance was based on several critical and major findings in the two investigators sites inspected, pertaining to trial management, and quality of the data”. “These issues are considered to have a major impact on the reporting of both efficacy and safety data, Thus, the credibility of the data is questioned and the benefit/risk cannot be assessed based on the currently submitted data”	422	30
*Mometasone furoate and formoterol fumarate*, in the treatment of asthma	Allergy and Asthma Proceedings, 2010, [105]	“A large number of critical and major findings were detected at the 2 other inspection sites”. “Due to the deficient monitoring and lack of adequate oversight the data reliability is questionable”	26	5
*Naproxcinod*, in the relief of signs and symptoms of osteoarthritis of the knee and hip	Osteoarthritis and Cartilage, 2010, [117]	“A routine GCP inspection for studies HCT 3012-X-301 and HCT 3012-X-303 in two investigator sites and one sponsor site (INS/GCP/2010/04) revealed a critical deviation in the site 804 of study 303: Several discrepancies between Data Listings and source documents (Case report form CRF) regarding the Womac scale values were detected”. “Due to the exclusion of data from patients at Site 061/069 (see Sect. 2.5.4.1.1), (failure to comply with GCP) the efficacy analyses of Studies HCT 3012-X-301 and HCT 3012-X-302 were repeated after sign-off of the reports. The re-analysis of the primary and key secondary efficacy data excluded the data on 36 patients from the ITT and PP populations. when data from Site 061/069 were excluded from Study HCT 3012-X-301 was that the naproxcinod 375 mg bid dose was no longer non-inferior to naproxen 500 mg bid at Week 13 for the WOMACTM pain subscale score”	38	5
*Inactivated influenza virus vaccine,* in the prevention of seasonal influenza in infants and children	The New England Journal of Medicine, 2011, [130]	“Results of the inspection revealed three critical findings and several major findings”. “The Inspection performed has put in evidence many relevant failures and the final report recommended not to accept the trial results due to the lack of compliance with GCP”. “Moreover, critical findings, questioning the validity of the PCR analysis, were discovered in the central laboratory that analysed all nasal swab samples collected in all study centres”. “The GCP Inspection found not only a lack of GCP compliance in the inspected sites but also identified a number of issues, regarding the clinical efficacy data from the pivotal trial, that were inaccurately stated in the first dossier submitted by the Applicant. This greatly impacts the reliability of overall study results”. “Moreover, from the efficacy point of view, the finding that none of the 152 children vaccinated at the site was reported to have ILI during the study period (several months) also questions the validity of overall efficacy data obtained in the trial. It is in fact noted that in total 4702 children were recruited in the pivotal trial, and 1114 of them had ILI (i.e. a 23.6% of all vaccinated children) during the trial period. Thus, the probability of not having any child with ILI out of the 152 vaccinated children in the site was: 0.000000000000000002 (= 2 × 10–18). The fact that the Applicant did include in the efficacy analyses data from this site, which are extremely unlikely to have occurred in reality, suggests that there was no appropriate supervision of the efficacy data by the Applicant”	244	10
*Peginesatide*, in the treatment of symptomatic anaemia associated with chronic kidney disease in adult patients undergoing dialysis	The New England Journal of Medicine, 2013, [82]	“Findings of the GCP inspection identified issues with regards to insufficient oversight and monitoring, irregularities in data handling, out of protocol dosing (adjustment) for the Epoetin groups and concerns with regards to the definition of the PP Population”. “However, GCP findings and uncertainties with regards to the PP Population are of major concern and question the overall data validity”. “This is of particular importance in non-inferiority”. “Efficacy in the maintenance treatment is not considered confirmed in the presence of GCP findings and uncertainties with regards to reliability of the data trials, where a clean study conduct is required to minimize the risk that true differences between treatments would not become apparent”	71	2
*Vaccine which contains hepatitis B surface antigen*, in the prevention of hepatitis B infection	Vaccine, 2013, [88]	“Due to the lack of quality system by the sponsor, the insufficient instruction with regard to IMP handling, storage and keeping the study blind as well as the incorrectness of the clinical study report in a number of fields, the data of trial DV2-HBV-17 are considered non-acceptable and the inspectors recommend not to use the data of trial DV2-HBV-17 in the context of the evaluation of the marketing authorisation application of Heplisav”. “The pivotal study, HBV-17 is not considered to contain sufficiently reliable data due to GCP issues”. “The findings related to the inappropriate quality assurance and quality control system are considered to be process-related and therefore apply for the entire study. Most likely, those findings also impact previous and on-going studies performed by the sponsor”	56	1
*Human fibrinogen and human thrombin*, in the treatment of bleeding during vascular surgery	Annals of Vascular Surgery, 2017, [76]	“The findings observed at the three sites inspected have a relevant impact on the quality and integrity of the data”. “Several GCP breaches where identified at all sites inspected including the sponsor and the affiliate and all of them along with the CRO critical performance contributed to the fact that data are not considered trustworthy”. “Some data included in the CSR could not be verified during the source data verification process as they were not in any of the source documents used at sites”. “This has an impact on data consistency and of course and on data integrity”. “At the sites discrepancies on surgical and non-surgical events were observed and a deficient management of safety documentation”	17	4
*Cediranib*, in the treatment of ovarian cancer	The Lancet, 2016, [91]	“A total of four study sites were inspected (of 62 participating study centers). The inspections revealed several GCP findings. The main issues were related to: - Oversight by the study sponsor which resulted in non-uniform conduct and reporting of data - The impact of post-unblinding activities on the reported data - Lack of available documentation of the appearance of test and control product meant the blinding process is not verifiable - The safety profile was considered incomplete, A routine inspection revealed issues, the impact of which can still not be fully assessed” “The most important remaining issues pertain to the reliability of the data and the integrity of the database. Based on an initial concordance analysis, there were important differences between investigator assessed PFS and PFS by BICR for 15 out of 36 evaluable patients in three inspected sites (i.e. 41% discordance), questioning the reliability of the adjudication of PFS”	193	15
*Trastuzumab*, in the treatment of certain forms of breast cancer and gastric (stomach) cancer	Annals of oncology (ESMO abstract), 2020, [132]	“The GCP inspection for the phase III TROIKA study (EudraCTnr: 2016–004019-11; EMA Inspection reference number: GCP/2019/022) revealed critical GCP non-compliance that affected the credibility of the data”. “Further to several critical findings during GCP-inspections and re-inspections, the applicant presented an updated clinical study report”. “In addition, as a result of the CAPA arising from the requested GCP inspection of the TROIKA study, neo-adjuvant subject data was 100% re-monitored and resubmitted, because substantial amounts of previously unreported safety data was discovered”. “Even if the discrepancies and lack of updating of reports had been partly sorted out, uncertainties remained regarding biosimilarity, due to concern regarding critical findings from the GCP inspections and re-inspections of the TROIKA study”	NA	NA
*Trastuzumab*, in the treatment of certain forms of breast cancer and gastric (stomach) cancer	Journal of clinical oncology (ASCO abstract), 2020, [86]	“The GCP inspection for the phase III TROIKA study (EudraCTnr: 2016–004019-11; EMA Inspection reference number: GCP/2019/022) revealed critical GCP non-compliance that affected the credibility of the data”. “Further to several critical findings during GCP-inspections and re-inspections, the applicant presented an updated clinical study report”. “In addition, as a result of the CAPA arising from the requested GCP inspection of the TROIKA study, neo-adjuvant subject data was 100% re-monitored and resubmitted, because substantial amounts of previously unreported safety data was discovered”. “Even if the discrepancies and lack of updating of reports had been partly sorted out, uncertainties remained regarding biosimilarity, due to concern regarding critical findings from the GCP inspections and re-inspections of the TROIKA study”	NA	NA
*Trastuzumab-duocarmazine* in the treatment of HER2-positive breast cancer	Annals of Oncology (ESMO abstract), 2021, [97]	“the inspection at the Singapore site was impaired by multiples administrative difficulties, and led to several critical findings, especially with regards to direct access to the electronic medical records of subjects”. “Due to important difficulties to access and control the study records, inspectors were of the opinion that these data could not be used in the context of a MAA”. “Moreover, when inspecting the Sponsor’s site, it was found that 11 other sites did not give full access to their electronic source data to the sponsor, but relied on paper documentation. These 12 investigational centres represented 17% of the study population, for which a very strong degree of uncertainty on the reliability of the data is identified”. “The GCP inspection identified critical findings on the sponsor’s lack of access to source data from 12 of the 78 clinical sites, representing 75 patients out of 473, leading to GCP uncompliant monitoring of these sites and potentially impairing data integrity”	NA	NA
*Trastuzumab-duocarmazine* in the treatment HER2-positive breast cancer	Journal of clinical oncology, 2024, [128]	“the inspection at the Singapore site was impaired by multiples administrative difficulties, and led to several critical findings, especially with regards to direct access to the electronic medical records of subjects”. “Due to important difficulties to access and control the study records, inspectors were of the opinion that these data could not be used in the context of a MAA”. “Moreover, when inspecting the Sponsor’s site, it was found that 11 other sites did not give full access to their electronic source data to the sponsor, but relied on paper documentation. These 12 investigational centres represented 17% of the study population, for which a very strong degree of uncertainty on the reliability of the data is identified”. “The GCP inspection identified critical findings on the sponsor’s lack of access to source data from 12 of the 78 clinical sites, representing 75 patients out of 473, leading to GCP uncompliant monitoring of these sites and potentially impairing data integrity”	3	1
*Daprodustat*, in the treatment of adult patients with symptoms of anaemia caused by chronic kidney disease	The New England Journal of Medicine, 2021, [120]	“In the study 200,808/ASCEND-ND and 200,807/ASCEND-D, three and eight sites were closed early due to GCP non-compliance issues, including suspected fraud at one and three sites, respectively; participants from these sites are excluded from the results presented.” “Data on 17 study participants (1 randomized) and 10 participants, respectively, were excluded from all analyses as valid informed consent was never obtained”. “For the ASCEND-D study, 88 subjects were randomised at three sites, 49 to the daprodustat arm and 39 to the rhEPO arm. There were no meaningful differences between the primary and adjusted analyses for the most relevant endpoints and study outcome of the ASCEND-D study. However, the detailed information on the nature of the issues at the three sites confirmed a high suspicion of fraud, including the alleged fabrication of data. The Applicant’s actions concerning the findings, i.e. closure of three sites, is considered acceptable. However, it is considered most correct to present the results with data from the closed sites excluded in the EPAR and SmPC. All tables and figures in the SmPC and/or EPAR containing data from the ASCEND-D study have been substituted with tables and figures with data from the 88 subjects affected by the site closure excluded”	120	17

*MA* meta-analysis, *GCP* good clinical practice, *CSR* clinical study report, *CHMP* Committee for Medicinal Products for Human Use, *IMP* investigational medicinal product, *AE* adverse events, *SAE* serious adverse events, *AFIRMM* Association Française pour les Initiatives dans la Recherche sur le Mastocyte et la Mastocytose, *CRO* contract research organisation, *NAM* National Agency for Medicines, *EMEA* European medicine Agencies, *MAL* acute myeloid leukemia, *ITT* intention to treat, *PP* per protocol, *WOMAC* Western Ontario and McMaster Universities Osteoarthritis Index, *ILI* influenza like illness, *PFS* progression-free survival, *BIRC* blinded independent central review, *EMA* European medicine Agencies, *CAPA* corrective and preventive action, *MAA* marketing authorisation applications, *rhEPO* recombinant human erythropoietin, *SmPC* summary of product characteristics, *EPAR* European public assessment report, *ESMO* European Society for Medical Oncology, *ASCO* American Society of Clinical Oncology

### Detailed inspection reports

Our first request for access to GCP inspection reports was made to the EMA on the 4th of December 2024. To date (September 12, 2025), we had no answers.

## Discussion

Among 285 EPARs concerning drugs that were not authorized in the European Union, we found 57 that mentioned a GCP inspection including 24 studies with issues affecting the reliability of the results, that were published in 26 different articles. None of those publications acknowledged the issues or were subsequently corrected after publication. While health authorities’ GCP inspection reports serve as a significant source of information, regulators cannot ensure their uptake by the publication ecosystem. This is particularly regrettable given that the medical journals are widely regarded as trusted sources of evidence. Many of their readers may be unfamiliar with the highly technical and detailed EPARs and instead rely on journal articles that could fail to provide the full picture for a given drug trial. Journals stand to gain significantly from such independent GCP inspections, which offer a level of scrutiny and depth that far exceeds what peer review alone can deliver. However, it is unclear who should communicate the results of the GCP inspection to the journals. Health regulatory agencies have no direct involvement with medical journals and have already published their reports. Authors and sponsors could be responsible, but potential conflicts of interest could limit their willingness to do so, and until there are clear guidelines on what to report, they cannot be held responsible.

We do not believe that all studies with potential reliability issues should be retracted. In some cases, the concerns may affect only a subset of outcomes or analyses (for example for daprodustat [70, 120], where exclusion of sites did not change the overall results). Nonetheless, journals have a responsibility to clearly indicate which parts of a study appear robust and which are questionable or irreparably flawed. The list provided in this paper may be an invaluable resource for achieving this objective.

While we adopted a comprehensive approach by examining all rejected or withdrawn EPARs, several limitations should be acknowledged. Despite the EMA’s efforts to promote transparency, EPARs often lack sufficient detail about GCP inspections, leaving the nature and impact of identified issues unclear in many cases. Due to our conservative data analysis, which only included explicit concerns and excluded “unclear” situations, we may have overlooked some problematic studies. Studies lacking data reliability assessment and GCP findings description should indeed be approached with caution. In these situations, a review of complete GPC inspection records could be beneficial. However, our requests for these reports have proven largely ineffective, with delays so long as to render the process practically unusable.

To facilitate the identification of EPARs that may contain GCP-related issues, we focused our analysis on refused and withdrawn EPARs. This approach may introduce selection bias and overestimate the frequency or severity of problems. It is reasonable to assume that studies supporting approved drugs are generally more robust, as these drugs are expected to be backed by more trustworthy evidence and less likely to have critical GCP findings impacting the data.

The clinical relevance of our findings may be limited, as these drugs are not approved for use in the European market. However, this limitation is mitigated by the facts that some of these products may be used off-label, some are marketed in other regions, all potentially serve as the basis for further research concerning broader pharmacological class and some may be ultimately marketed in European Union following a new market application. Furthermore, our focus was on the reliability of the published studies’ records, which is relevant regardless of drug approval status. It is also important to note that our focus was on drugs undergoing market authorisation submission, thus our analysis concerned mostly large pivotal trials sponsored by pharmaceutical companies.

Bearing these limitations in mind, our study aligns with and replicates findings from the previous analysis of FDA data by Seife [6] with several subtle differences. Both studies found that violations of GCP identified by health authorities are almost never reported in peer-reviewed literature. In addition, these analyses offer complementary insights; Seife’s analysis provides detailed observations, but sometimes lacks clarity on the exact impact of GPC violations on study results [6]. As illustrated in our study, health authorities might have identified “critical findings” (EMA) or “official action indicated” (the most severe classification by the FDA [6]) that do not affect the data reliability or that are not clinically relevant. Without direct access to GPC inspections, our analysis is less precise, but CHMP’s assessment of impact on study results helps identify studies critically needing mention of inspections in their publication. In practice, GCP inspections provide site-level information on trial conduct and data integrity, whereas EPARs reflect the overall regulatory assessment of the application taking GCP findings in account. Both GCP reports and expert committee analyses are valuable for end users.

Interestingly, EPARs have been the subject of some previous research [133, 134], but we found no studies that systematically used them to extract information about GCP inspections. Our study and that of C. Seife detailed some inspections performed by the FDA and EMA, but reports from other national regulatory agencies could also be used to identify additional studies with reliability issues.

All these results show a clear gap between health authorities’ data and medical literature. Journal processes appear suboptimal, particularly for correcting the record. There is considerable data on the limitations of post-publication peer review as currently implemented [135–138]. Journals often enforce editorial policies that set word or time constraints following the initial publication of an article [139]. Journals occasionally offer online comments but those often lack visibility. We did not systematically search for such comments, but we found two studies (about anamorelin [126] and inactivated influenza virus vaccine for children [130]) that were subject of online comments about the GCP inspection [140, 141]. These comments explicitly pointed out the issues with data reliability. However, no correction was made to the studies despite those comments. Consequently, post-publication peer review is underdeveloped and often ineffective. In the absence of concrete mechanisms for accounting for GCP inspections outcomes in the medical literature, we believe that studies like this one and initiatives like PubPeer (a site dedicated to post publication peer review) [13] are essential to raise awareness until systemic action is implemented. For example, systematic search for regulatory evaluation and inspection reports could also be implemented in systematic reviews and meta-analyses.

## Conclusions

This systematic analysis of GCP inspections, as documented in the EPARs of drugs not authorized in the European Union, identified several studies published in the medical literature that warrant correction. Future research projects should analyse full GCP inspection reports and extend this line of inquiry to drugs approved by the EMA.

## Supplementary Information

Additional file 1. supplementary methods

Additional file 2: Supplementary Tables: Table S1: List of EPAR and description of good clinical practice inspection. findings. Legend: * Sponsor clinical number; GVHD = graft versus host disease; ±  = EudraCT trial. number; ‡ = National Clinical Trial;—= not mentioned; NA = Not available. Table S2: EPAR and publications paired, description of the relevance of the good clinical practice inspection findings on the data reliability and diffusion of the studies in the literature. Legend: * = meeting abstract; GVHD = graft versus host disease; ESMO = European Society for Medical Oncology; ASCO = American Society of Clinical Oncology. Table S3: List of unpublished studies and impact of the good clinical practice inspection findings on the data reliability. Legend: * Sponsor clinical number; ±  = EudraCT trial number; ‡ = National Clinical Trial; NA = Not available

## Data Availability

Data and code to reproduce the analysis are available on the Open Science Framework ([https://osf.io/pa9fq/files/osfstorage](https:/osf.io/pa9fq/files/osfstorage) (10)). EPARs used for the study are cited via the EMA link.

## Abbreviations

MA: Meta-analysis
GCP: Good Clinical Practice
CSR: Clinical study report
CHMP: Committee for Medicinal Products for Human Use
IMP: Investigational medicinal product
EMA: European Medicines Agencies
FDA: Food and Drug Administration
EPAR: European Public Assessment Report
IQR: Interquartile range
