# Supplementary material for: Concordance between European medicine agency good clinical practice inspections and medical literature: a meta-research survey
Source: BMC Med. 2025 Dec 3;23:674. doi: 10.1186/s12916-025-04499-9 (PMC12676876; doi:10.1186/s12916-025-04499-9)
Supplement: Supplementary file 1 — Additional file 1. supplementary methods [file 12916_2025_4499_MOESM1_ESM.docx]

**Additional file 1: supplementary methods:**

***Patient involvement***

Patients were not involved in formulating the research question or the outcome measures, nor did they participate in the design and implementation of the study. The findings of this project will be presented at the first meeting of the external committee for RestoRes (Research integrity in biomedical research), funded by the French Agence Nationale de la Recherche under grant agreement ANR-23-CE36-0006. This committee, which includes patient representatives and citizens, will be consulted to discuss the implications of the findings and determine the concrete actions to be implemented following this research project.

***EMA definitions***

Critical findings are defined as “conditions, practices, or processes that adversely affect the rights, safety or wellbeing of the subjects and/or the quality and integrity of data”. Major findings are defined as “conditions practices or processes that might adversely affect the rights, safety or wellbeing of the subjects and/or the quality and integrity of data. Major observation are serious deficiencies and are direct violation of GCP principles”. Minor deviations are defined as “conditions, practices or processes that would not be expected to adversely affect the right, safety or wellbeing of the subjects and/or the quality and integrity of data”

***Search strategy***

The EPARs were searched under "Clinical efficacy" and "Main studies" for any EU Clinical Trials Register or ClinicalTrials.gov trial identification number or study number used by the sponsor. Then, we searched in Google Scholar and study registries for the clinical studies published in medical journals using the identification number. We considered both studies published as articles or conference presentations and recorded them separately. In the absence of an identification number, we searched in free text the descriptive elements of the study using the drug's name (commercial or international non-proprietary name), study design regarding randomization, comparator, the presence of blinding (single or double), and the medical indication (disease name with stage if specified) in the Google search engine.
