# Supplementary material for: Concordance between European medicine agency good clinical practice inspections and medical literature: a meta-research survey
Source: BMC Med. 2025 Dec 3;23:674. doi: 10.1186/s12916-025-04499-9 (PMC12676876; doi:10.1186/s12916-025-04499-9)
Supplement: Supplementary file 2 — Additional file 2: Supplementary Tables: Table S1: List of EPAR and description of good clinical practice inspection. findings. Legend: * Sponsor clinical number; GVHD = graft versus host disease; ± = EudraCT trial. number; ‡ = National Clinical Trial;—= not mentioned; NA = Not available. Table S2: EPAR and publications paired, description of the relevance of the good clinical practice inspection findings on the data reliability and diffusion of the studies in the literature. Legend: * = meeting abstract; GVHD = graft versus host disease; ESMO = European Society for Medical Oncology; ASCO = American Society of Clinical Oncology. Table S3: List of unpublished studies and impact of the good clinical practice inspection findings on the data reliability. Legend: * Sponsor clinical number; ± = EudraCT trial number; ‡ = National Clinical Trial; NA = Not available [file 12916_2025_4499_MOESM2_ESM.docx]

**Additional file 2: Supplementary Tables:**

Table S1: List of EPAR and description of good clinical practice inspection

findings.

Legend: * Sponsor clinical number; GVHD = graft versus host disease; ± = EudraCT trial

number; ‡ = National Clinical Trial; - = not mentioned; NA= Not available.

Table S2: EPAR and publications paired, description of the relevance of the

good clinical practice inspection findings on the data reliability and diffusion of the studies in

the literature.

Legend: * = meeting abstract; GVHD = graft versus host disease; ESMO = European Society

for Medical Oncology; ASCO = American Society of Clinical Oncology.

Table S3: List of unpublished studies and impact of the good clinical practice

inspection findings on the data reliability.

Legend: * Sponsor clinical number; ± = EudraCT trial number; ‡ = National Clinical Trial;

NA= Not available

**Table S1**: List of EPAR and description of good clinical practice inspection findings.

| **Drug (commercial, used in EPAR)** | **Drug (International non-proprietary name)** | **Deviation** | | | **Concerns about** | | | **Number of critical deviation** | **Related studies** | **Number of articles related** | **EPAR reference** |
| --- | --- | --- | --- | --- | --- | --- | --- | --- | --- | --- | --- |
|  |  | **Critical** | **Major** | **Minor** | **Efficacy** | **Safety** | **Ethic** |  |  |  |  |
| Alpheon | Interferon alfa-2a | - | - | - | - | - | - | NA | BP-IFN-002* | 0 | (19) |
| Mycograb | Efungumab | Yes | Yes | - | Yes | Yes | Yes | NA | NA | 1 | (15) |
| Zeftera | Ceftobiprole | - | - | - | Yes | Yes | - | NA | 2004-001662-41^±^ ; 2005-002301-23^±^ | 1 | (28) |
| Sumatriptan Galpharm | Sumatriptan | - | - | - | - | - | - | NA | 03-115* | 0 | (32) |
| Istodax | Romidepsin | - | Yes | - | Yes | - | - | 0 | GPI-06-0002* | 1 | (34) |
| Labazenit | budesonide / salmeterol | Yes | - | - | Yes | - | - | NA | BUSAL SS071* ; BUSAL III-02-01* | 0 | (38) |
| Masican | Masitinib | Yes | Yes | - | Yes | Yes | - | 23 | AB07001* | 1 | (41) |
| Reasanz | Serelaxin | - | Yes | - | - | - | - | 0 | NCT00520806^‡^ | 1 | (42) |
| Heparesc | Human heterologous liver cells | Yes | Yes | - | Yes | Yes | - | 11 | NCT00718627^‡^ | 1 | (47) |
| Human IGG1 monoclonal antibody specific for human interleukin-1 alpha XBiotech | Human IgG1 monoclonal antibody specific for human interleukin-1 alpha | - | - | - | - | - | - | NA | 2014-000550-12^±^ | 1 | (55) |
| Adlumiz | Anamorelin | - | - | - | Yes | Yes | - | NA | NCT01387269^‡^ ; NCT01387282^‡^ | 1 | (54) |
| Masipro | Masitinib | Yes | Yes | Yes | Yes | Yes | - | NA | NCT00814073^‡^ | 1 | (56) |
| Xeljanz | Tofacitinib | Yes | Yes | - | - | - | - | NA | NCT00814307^‡^;  NCT00814307^‡^; NCT00847613^‡^ | 3 | (40) |
| Alsitek | Masitinib | Yes | Yes | Yes | Yes | Yes | - | 4 | NCT02588677^‡^ | 2 | (60) |
| Eladynos | Abaloparatide | Yes | - | - | - | - | - | NA | NCT01343004^‡^ | 1 | (59) |
| Gamifant | Emapalumab | Yes | Yes | - | Yes | Yes | Yes | NA | NCT01818492^‡^ | 1 | (66) |
| Nouryant | Istradefylline | - | - | - | - | - | - | NA | NCT00456586^‡^; NCT00456794^‡^; 6002-US-013*; 6002-0608*; NCT00955526^‡^; 6002-US-018*; NCT00199394^‡^; NCT01968031^‡^ | 6 | (68) |
| Arxxant | Ruboxistaurin | - | - | - | - | - | - | NA | NCT00604383^‡^ | 1 | (16) |
| Cerepro | Herpes simplex virus thymidine kinase gene | - | - | - | - | - | - | NA | Study 903* | 1 | (17) |
| Retisert | Fluocinolone acetonide | - | - | - | - | - | - | NA | BLP 415-002* | 1 | (18) |
| Kiacta | Eprodisate disodium | - | - | - | Yes | - | - | NA | NCT00035334^‡^ | 1 | (20) |
| Lenalidomide Celgene Europe | Lenalidomide | - | - | - | Yes | Yes | - | NA | NCT00065156^‡^ | 1 | (22) |
| Aflunov | A/VietNam/1194/2004 vaccine | - | - | - | Yes | Yes | Yes | NA | 2006-005428-18^±^ | 0 | (21) |
| Vekacia | Ciclosporin | - | - | - | - | - | - | NA | NCT00328653^‡^ | 1 | (23) |
| Nenad | Lisuride | - | - | - | - | - | - | NA | TULIP-IIb* | 0 | (24) |
| Oncophage | Vitespen | - | Yes | - | - | - | - | 0 | NCT00033904^‡^ | 1 | (25) |
| Repaglinide Sun | Repaglinide | - | Yes | - | - | - | - | 0 | PKD_08_059* | 0 | (27) |
| Tyvaso | Treprostinil sodium | Yes | Yes | - | Yes | Yes | - | 6 | NCT00147199^‡^ | 1 | (26) |
| Zenhale | Mometasone furoate and formoterol fumarate | Yes | Yes | - | - | - | - | NA | NCT00383240^‡^ | 1 | (29) |
| Beprana | Naproxcinod | Yes | - | - | Yes | - | - | NA | NCT00542555^‡^;  NCT00504127^‡^;  NCT00541489^‡^ | 3 | (30) |
| Luveniq | Voclosporin | Yes | Yes | - | Yes | Yes | - | NA | 2006-006543-31^±^; 2006-006544-66^±^ | 1 | (31) |
| Fluad Paediatric | Inactivated influenza (flu) virus strains | Yes | Yes | - | Yes | Yes | - | 3 | NCT00644059^‡^ | 1 | (33) |
| Loulla | Mercaptopurine | - | - | - | - | - | - | NA | NA | 0 | (36) |
| Jenzyl | Ridaforolimus | - | Yes | - | Yes | - | - | 0 | NCT00538239^‡^ | 1 | (35) |
| Omontys | Peginesatide | - | - | - | Yes | - | - | NA | NCT00597753^‡^;  NCT00597584^‡^ | 1 | (39) |
| OraNera | Autologous oral mucosal epithelial cells | Yes | Yes | - | - | Yes | - | NA | 2007-A00270-53^±^ | 1 | (37) |
| Heplisav | Vaccine which contains hepatitis B surface antigen | - | - | - | Yes | Yes | - | NA | NCT00985426^‡^ | 1 | (43) |
| Neocepri | Folic acid | - | - | - | - | - | - | NA | NA | 0 | (46) |
| Vynfinit | Vintafolide | - | - | - | - | - | - | NA | NCT00722592^‡^ | 1 | (45) |
| Folcepri | Etarfolatide | - | - | - | - | - | - | NA | NA | 0 | (44) |
| Veraseal | Human fibrinogen and human thrombin | Yes | - | - | Yes | Yes | - | NA | NCT01754480^‡^ | 1 | (48) |
| Kyndrisa | Drisapersen | - | - | - | - | Yes | - | NA | NCT01480245^‡^ | 0 | (50) |
| Opsiria | Sirolimus | - | - | - | - | - | - | 0 | NCT01358266^‡^ | 1 | (49) |
| Zemfirza | Cediranib | - | - | - | Yes | Yes | - | NA | NCT00532194^‡^ | 1 | (51) |
| Graspa | Eryaspase | - | Yes | - | - | - | Yes | 0 | NCT01518517^‡^ | 1 | (53) |
| Efgratin | Pegfilgrastim | - | Yes | Yes | - | - | - | 0 | 2013-003166-14^±^ | 1 | (52) |
| Qinprezo | Vosaroxin | - | Yes | Yes | - | - | - | 0 | NCT01191801^‡^ | 1 | (57) |
| Qizenday | Biotin | - | Yes | - | Yes | - | - | 0 | 2013-002113-35^±^ | 1 | (58) |
| Nuzyra | Omadacycline | - | - | - | - | - | - | NA | NCT02378480^‡^; NCT02531438^‡^ | 2 | (61) |
| Idhifa | Enasidenib | Yes | Yes | - | - | - | - | NA | NCT01915498^‡^ | 2 | (63) |
| Luxceptar | T-cells from the transplant donor, specially treated to reduce the risk of GVHD | - | Yes | - | - | - | - | 0 | NCT01794299^‡^ | 2 | (62) |
| Doxorubicin Hydrochloride Tillomed | Doxorubicin | Yes | Yes | - | Yes | - | - | 2 | PLCL 200 17* | 0 | (64) |
| Sondelbay | Teriparatide | - | - | - | - | - | - | NA | 0425-17* | 0 | (65) |
| Upkanz | Deferiprone | - | Yes | Yes | - | - | - | 0 | NCT01741532^‡^ | 1 | (67) |
| Tuznue | Trastuzumab | Yes | Yes | - | Yes | Yes | - | NA | 2016-004019-11^±^ | 2 | (69) |
| Jivadco | Trastuzumab-duocarmazine | Yes | Yes | - | Yes | Yes | - | NA | NCT03262935^‡^ | 2 | (71) |
| Jesduvroq | Daprodustat | - | - | - | - | - | - | NA | NCT02876835^‡^;   NCT02879305^‡^ | 2 | (70) |

Table legend: GVHD = graft versus host disease; * Sponsor clinical number; ± = EudraCT trial number; ‡ = National Clinical Trial; - = not mentioned; NA= Not available.

**Table S2:** EPAR and publications paired, description of the relevance of the good clinical practice inspection findings on the data reliability and diffusion of the studies in the literature.

| **Drug (commercial) (EPAR reference)** | **Drug (International non-proprietary name) (publication reference)** | **PMID** | **DOI** | **Journal** | **Number of citations** | **Citation by Meta-analysis** | **Data Reliability** |
| --- | --- | --- | --- | --- | --- | --- | --- |
| Mycograb (15) | Efungumab (110) | 16619152 | 10.1086/503428 | Clinical Infectious Diseases | 227 | 2 | Not affected |
| Zeftera (28) | Ceftobiprole (108) | 18225981 | 10.1086/526527 | Clinical Infectious Diseases | 208 | 12 | Affected |
| Istodax (34) | Romidepsin (77) | 22271479 | 10.1200/JCO.2011.37.4223 | Journal of clinical oncology | 533 | 6 | Unknown |
| Masican (41) | Masitinib (72) | 25122671 | [10.1093/annonc/mdu237](https://doi.org/10.1093/annonc/mdu237) | Annals of oncology | 54 | 6 | Affected |
| Reasanz (42) | Serelaxin (125) | 23141816 | [10.1016/S0140-6736(12)61855-8](https://doi.org/10.1016/s0140-6736(12)61855-8) | The Lancet | 702 | 3 | Not affected |
| Heparesc (47) | human heterologous liver cells (99) | 29027067 | [10.1007/s10545-017-0097-4](https://doi.org/10.1007/s10545-017-0097-4) | Journal of Inherited Metabolic Disease | 14 | 0 | Affected |
| Human IGG1 monoclonal antibody specific for human interleukin-1 alpha XBiotech (55) | human IgG1 monoclonal antibody specific for human interleukin-1 alpha (85) | 28094194 | [10.1016/S1470-2045(17)30006-2](https://doi.org/10.1016/s1470-2045(17)30006-2) | The Lancet Oncology | 120 | 1 | Unknown |
| Adlumiz (54) | Anamorelin (126) | 26906526 | [10.1016/S1470-2045(15)00558-6](https://doi.org/10.1016/s1470-2045(15)00558-6) | The Lancet Oncology | 415 | 9 | Affected |
| Masipro (56) | Masitinib (96) | 28069279 | [10.1016/S0140-6736(16)31403-9](https://doi.org/10.1016/s0140-6736(16)31403-9) | The Lancet | 93 | 2 | Affected |
| Xeljanz (40) | Tofacitinib (83) | 22873530 | [10.1056/NEJMoa1109071](https://doi.org/10.1056/nejmoa1109071) | The New England Journal of Medicine | 742 | 44 | Not affected |
| Xeljanz (40) | Tofacitinib (124) | [26530039](https://pubmed.ncbi.nlm.nih.gov/26530039) | [10.1186/s13075-015-0825-9](https://doi.org/10.1186%2Fs13075-015-0825-9) | Arthritis Research & Therapy | 51 | 4 | Not affected |
| Xeljanz (40) | Tofacitinib (129) | 23348607 | [10.1002/art.37816](https://doi.org/10.1002/art.37816) | Arthritis & Rheumatism | 455 | 42 | Affected |
| Alsitek (60) | Masitinib (104) | 31280619 | [10.1080/21678421.2019.1632346](https://doi.org/10.1080/21678421.2019.1632346) | Amyotrophic Lateral Sclerosis and Frontotemporal Degeneration | 135 | 1 | Affected |
| Alsitek (60) | Masitinib (103) | 34457038 | [10.1177/17562864211030365](https://doi.org/10.1177/17562864211030365) | [Therapeutic Advances in Neurological Disorders](https://journals.sagepub.com/home/TAN) | 38 | 0 | Affected |
| Eladynos (59) | Abaloparatide (100) | 27533157 | [10.1001/jama.2016.11136](https://doi.org/10.1001/jama.2016.11136) | JAMA | 571 | 25 | Affected |
| Gamifant (66) | Emapalumab (95) | 32374962 | [10.1056/NEJMoa1911326](https://doi.org/10.1056/nejmoa1911326) | The New England Journal of Medicine | 319 | 0 | Affected |
| Nouryant (68) | Istradefylline (93) | 18306243 | [10.1002/ana.21315](https://doi.org/10.1002/ana.21315) | Annals of Neurology | 284 | 8 | Unknown |
| Nouryant (68) | Istradefylline (121) | 18519872 | [10.1212/01.wnl.0000313834.22171.17](https://doi.org/10.1212/01.wnl.0000313834.22171.17) | Neurology | 145 | 6 | Unknown |
| Nouryant (68) | Istradefylline (84) | 18831530 | [10.1002/mds.22095](https://doi-org.proxy.insermbiblio.inist.fr/10.1002/mds.22095) | Movement Disorders | 167 | 8 | Unknown |
| Nouryant (68) | Istradefylline (101) | 20629136 | [10.1002/mds.23107](https://doi-org.proxy.insermbiblio.inist.fr/10.1002/mds.23107) | Movement Disorders | 141 | 8 | Unknown |
| Nouryant (68) | Istradefylline (102) | [23483627](https://pubmed.ncbi.nlm.nih.gov/23483627) | [10.1002/mds.25418](https://doi.org/10.1002%2Fmds.25418) | Movement Disorders | 167 | 6 | Unknown |
| Nouryant (68) | Istradefylline (112) | 22000279 | [10.1016/j.parkreldis.2011.09.023](https://doi.org/10.1016/j.parkreldis.2011.09.023) | Parkinsonism Related disoders | 88 | 8 | Unknown |
| Arxxant (16) | Ruboxistaurin (78) | 18708615 | [10.1167/iovs.08-2473](https://doi.org/10.1167/iovs.08-2473) | Investigative Ophthalmology & Visual Science | 64 | 0 | Unknown |
| Cerepro (17) | Herpes simplex virus thymidine kinase gene (87) | 15509514 | [10.1016/j.ymthe.2004.08.002](https://doi.org/10.1016/j.ymthe.2004.08.002) | Molecular Therapy | 298 | 5 | Affected |
| Retisert (18) | Fluocinolone acetonide (111) | 20079922 | [10.1016/j.ophtha.2009.11.027](https://doi.org/10.1016/j.ophtha.2009.11.027) | Ophtalmology | 110 | 2 | Unknown |
| Kiacta (20) | Eprodisate disodium (80) | 17554116 | [10.1056/NEJMoa065644](https://doi.org/10.1056/nejmoa065644) | The New England Journal of Medicine | 162 | 1 | Affected |
| Lenalidomide Celgene Europe (22) | Lenalidomide (94) | 17021321 | [10.1056/NEJMoa061292](https://doi.org/10.1056/nejmoa061292) | The New England Journal of Medicine | 1009 | 6 | Affected |
| Vekacia (23) | Ciclosporin (92) | 37872059 | [10.1016/j.clinthera.2023.09.022](https://doi.org/10.1016/j.clinthera.2023.09.022) | [Clinical Therapeutics](https://www-sciencedirect-com.proxy.insermbiblio.inist.fr/journal/clinical-therapeutics) | 2 | 0 | Unknown |
| Oncophage (25) | Vitespen (131) | 18602688 | [10.1016/S0140-6736(08)60697-2](https://doi.org/10.1016/s0140-6736(08)60697-2) | The Lancet | 263 | 6 | Unknown |
| Tyvaso (26) | Treprostinil sodium (98) | 20430262 | [10.1016/j.jacc.2010.01.027](https://doi.org/10.1016/j.jacc.2010.01.027) | Journal of the American College of Cardiology | 422 | 30 | Affected |
| Zenhale (29) | Mometasone furoate and formoterol fumarate (105) | 20678306 | [10.2500/aap.2010.31.3364](https://doi.org/10.2500/aap.2010.31.3364) | Allergy and Asthma Proceedings | 26 | 5 | Affected |
| Beprana (30) | Naproxcinod (117) | 20202489 | [10.1016/j.joca.2009.12.013](https://doi.org/10.1016/j.joca.2009.12.013) | Osteoarthritis and Cartilage | 38 | 5 | Affected |
| Beprana (30) | Naproxcinod (118) | 20828790 | [10.1016/j.semarthrit.2010.06.002](https://doi.org/10.1016/j.semarthrit.2010.06.002) | [Seminars in Arthritis and Rheumatism](https://www-sciencedirect-com.proxy.insermbiblio.inist.fr/journal/seminars-in-arthritis-and-rheumatism) | 33 | 4 | Unknown |
| Beprana (30) | Naproxcinod (73) | 20722026 | [10.1002/art.27694](https://doi.org/10.1002/art.27694) | Arthritis & Rheumatology | 28 | 3 | Not affected |
| Luveniq (31) | Voclosporin (114) | NA | NA (Investigative Ophthalmology & Visual Science April 2009, Vol.50, issue 13) | Investigative Ophthalmology & Visual Science* | NA | NA | Unknown |
| Fluad Paediatric (33) | Inactivated influenza (flu) virus strains (130) | 21995388 | [10.1056/NEJMoa1010331](https://doi.org/10.1056/nejmoa1010331) | The New England Journal of Medicine | 244 | 10 | Affected |
| Jenzyl (35) | Ridaforolimus (81) | 23715582 | [10.1200/JCO.2012.45.5766](https://doi.org/10.1200/jco.2012.45.5766) | Journal of Clinical Oncology | 179 | 10 | Not affected |
| Omontys (39) | Peginesatide (82) | 23343061 | [10.1056/NEJMoa1203165](https://doi.org/10.1056/nejmoa1203165) | The New England Journal of Medicine | 71 | 2 | Affected |
| OraNera (37) | Autologous oral mucosal epithelial cells (75) | 22064987 | [10.1167/iovs.11-7744](https://doi.org/10.1167/iovs.11-7744) | Investigative ophtalmology & visual science | 133 | 0 | Not affected |
| Heplisav (43) | vaccine which contains hepatitis B surface antigen (88) | 23727422 | [10.1016/j.vaccine.2013.05.067](https://doi.org/10.1016/j.vaccine.2013.05.067) | Vaccine | 56 | 1 | Affected |
| Vynfinit (45) | Vintafolide (106) | 24127448 | [10.1200/JCO.2013.49.7685](https://doi.org/10.1200/jco.2013.49.7685) | Journal of Clinical Oncology | 151 | 4 | Not affected |
| Veraseal (48) | Human fibrinogen and human thrombin (76) | 28647631 | [10.1016/j.avsg.2017.06.043](https://doi.org/10.1016/j.avsg.2017.06.043) | Annals of Vascular Surgery | 17 | 4 | Affected |
| Opsiria (49) | Sirolimus (107) | 27692526 | [10.1016/j.ophtha.2016.07.029](https://doi.org/10.1016/j.ophtha.2016.07.029) | [Ophthalmology](https://www.sciencedirect.com/journal/ophthalmology) | 60 | 0 | Not affected |
| Zemfirza (51) | Cediranib (91) | 27025186 | [10.1016/S0140-6736(15)01167-8](https://doi.org/10.1016/s0140-6736(15)01167-8) | The Lancet | 193 | 15 | Affected |
| Graspa (53) | Eryaspase (74) | NA | [10.1182/blood.V126.23.3723.3723](https://doi.org/10.1182/blood.V126.23.3723.3723) | ASH (Volume 126, Issue 23, 3 December 2015, Page 3723 ; Blood)* | NA | NA | Not affected |
| Efgratin (52) | Pegfilgrastim (89) | 30727980 | [10.1186/s12885-019-5329-6](https://doi.org/10.1186/s12885-019-5329-6) | BMC Cancer | 5 | 1 | Not affected |
| Qinprezo (57) | Vosaroxin (113) | [26234174](https://pubmed.ncbi.nlm.nih.gov/26234174/) | [10.1016/S1470-2045(15)00201-6](https://doi.org/10.1016/S1470-2045(15)00201-6) | Lancet Oncology | 124 | 2 | Not affected |
| Qizenday (58) | Biotin (127) | 27589059 | [10.1177/1352458516667568](https://doi.org/10.1177/1352458516667568) | Multiple Sclerosis Journal | 221 | 0 | Not affected |
| Nuzyra (61) | Omadacycline (109) | 30726689 | [10.1056/NEJMoa1800170](https://doi.org/10.1056/nejmoa1800170) | The New England Journal of Medicine | 119 | 7 | Not affected |
| Nuzyra (61) | Omadacycline (123) | 30726692 | [10.1056/NEJMoa1800201](https://doi.org/10.1056/nejmoa1800201) | The New England Journal of Medicine | 155 | 4 | Not affected |
| Idhifa (63) | Enasidenib (122) | 28588020 | [10.1182/blood-2017-04-779405](https://doi.org/10.1182/blood-2017-04-779405) | Blood | 1095 | 3 | Not affected |
| Idhifa (63) | Enasidenib (79) | 34427990 | 10.1002/cam4.4182 | Cancer med | 6 | 0 | Not affected |
| Luxceptar (62) | T-cells from the transplant donor, specially treated to reduce the risk of GVHD (116) | NA | [10.1182/blood-2018-99-119086](https://doi.org/10.1182/blood-2018-99-119086) | Blood | NA | NA | Not affected |
| Luxceptar (62) | T-cells from the transplant donor, specially treated to reduce the risk of GVHD (115) | 32047237 | [10.1038/s41375-020-0733-0](https://doi.org/10.1038/s41375-020-0733-0) | Leukemia | 19 | 0 | Not affected |
| Upkanz (67) | Deferiprone (90) | 31202468 | [10.1016/S1474-4422(19)30142-5](https://doi.org/10.1016/s1474-4422(19)30142-5) | Lancet Neurology | 90 | 1 | Not affected |
| Tuznue (69) | Trastuzumab (132) | NA | [10.1016/j.annonc.2020.08.288](https://doi.org/10.1016/j.annonc.2020.08.288) | Annals of oncology (ESMO abstract)* | NA | NA | Affected |
| Tuznue (69) | Trastuzumab (86) | NA | [10.1200/JCO.2020.38.15_suppl.579](https://doi.org/10.1200/JCO.2020.38.15_suppl.579) | Journal of clinical oncology (ASCO abstract)* | NA | NA | Affected |
| Jivadco (71) | Trastuzumab-duocarmazine (97) | NA | [10.1016/j.annonc.2021.08.2088](https://doi.org/10.1016/j.annonc.2021.08.2088) | Annals of Oncology (ESMO abstract)* | NA | NA | Affected |
| Jivadco (71) | trastuzumab-duocarmazine (128) | 39442070 | [10.1200/JCO.24.00529](https://doi.org/10.1200/jco.24.00529) | Journal of clinical oncology | 3 | 1 | Affected |
| Jesduvroq (70) | Daprodustat (119) | 34739196 | [10.1056/NEJMoa2113380](https://doi.org/10.1056/nejmoa2113380) | The New England Journal of Medicine | 138 | 15 | Not affected |
| Jesduvroq (70) | Daprodustat (120) | 34739194 | [10.1056/NEJMoa2113379](https://doi.org/10.1056/nejmoa2113379) | The New England Journal of Medicine | 120 | 17 | Affected |

Table legend: * = meeting abstract; GVHD = graft versus host disease; ESMO = European Society for Medical Oncology; ASCO = American Society of Clinical Oncology.

**Table S3:** List of unpublished studies and impact of the good clinical practice inspection findings on the data reliability.

| **Drug (commercial)** | **Drug (International non-proprietary name)** | **EPAR reference** | **Related study** | **Data Reliability** |
| --- | --- | --- | --- | --- |
| Alpheon | interferon alfa-2a | (19) | BP-IFN-002* | Not affected |
| Zeftera | ceftobiprole | (28) | 2004-001662-41± | Affected |
| Sumatriptan Galpharm | sumatriptan | (32) | 03-115* | Affected |
| Labazenit | budesonide / salmeterol | (38) | BUSAL SS071* | Not affected |
| Labazenit | budesonide / salmeterol | (38) | BUSAL III-02-01* | Unknown |
| Nouryant | istradefylline | (68) | NCT00199394‡ | Unknown |
| Nouryant | istradefylline | (68) | NCT01968031‡ | Unknown |
| Aflunov | A/VietNam/1194/2004 vaccine | (21) | [2006-005428-18](https://www.clinicaltrialsregister.eu/ctr-search/search?query=2006-005428-18)± | Affected |
| Nenad | lisuride | (24) | TULIP-IIb* | Unknown |
| Repaglinide Sun | repaglinide | (27) | PKD_08_059* | Unknown |
| Loulla | mercaptopurine | (36) | NA | Affected |
| Neocepri | folic acid | (46) | NA | Not affected |
| Folcepri | etarfolatide | (44) | NA | Not affected |
| Kyndrisa | drisapersen | (50) | NCT01480245‡ | Not affected |
| Doxorubicin Hydrochloride Tillomed | doxorubicin | (64) | PLCL 200 17* | Affected |
| Sondelbay | teriparatide | (65) | 0425-17* | Affected |

Table legend: * Sponsor clinical number; ± = EudraCT trial number; ‡ = National Clinical Trial; NA= Not available.
